# Faster postnatal decline in hepatic erythropoiesis than granulopoiesis in human newborns

**DOI:** 10.3389/fped.2025.1572836

**Published:** 2025-05-20

**Authors:** Petra Janovska, Kristina Bardova, Zuzana Prouzova, Ilaria Irodenko, Tatyana Kobets, Eliska Haasova, Lenka Steiner Mrazova, Viktor Stranecky, Stanislav Kmoch, Martin Rossmeisl, Petr Zouhar, Jan Kopecky

**Affiliations:** ^1^ Laboratory of Adipose Tissue Biology, Institute of Physiology of the Czech Academy of Sciences, Prague, Czechia; ^2^ Department of Pathology, 3rd Faculty of Medicine, Charles University, University Hospital Kralovske Vinohrady, Prague, Czechia; ^3^ Department of Pathology, 1st Faculty of Medicine, Charles University, General University Hospital, Prague, Czechia; ^4^ Metabolomics Service Laboratory, Institute of Physiology of the Czech Academy of Sciences, Prague, Czechia; ^5^ Faculty of Science, Charles University, Prague, Czechia; ^6^ Research Unit for Rare Diseases, Department of Pediatrics and Inherited Metabolic Disorders, 1st Faculty of Medicine, Charles University, Prague, Czechia

**Keywords:** newborn, postnatal, haematopoiesis, RNA-Seq, gestation, human, liver

## Abstract

**Background:**

During human foetal development, the liver is the primary site of blood cell production, but this activity declines in the third trimester and postnatally as haematopoiesis shifts to bone marrow. In humans, this postnatal decline is not well characterized due to the scarcity of appropriate samples.

**Objective:**

To characterize the effect of (i) gestational age at birth and (ii) length of survival after birth on hepatic haematopoiesis across various cell lineages involved.

**Methods:**

Liver autopsy samples from 25 born-alive infants, predominantly extremely preterm newborns who died mainly between 1 day and 3 weeks after birth, were analysed. Haematopoiesis was characterized using immunohistochemical staining of established cell type-specific protein markers. RNA-sequencing data from our previous study using the same samples were also explored.

**Results:**

Haematopoiesis negatively correlates with both the duration of prenatal development and the length of postnatal survival. The effect of these two factors varies across different haematopoietic cell lineages. Prenatally and early postnatally, erythropoietic cells dominated hepatic haematopoiesis but were rapidly suppressed within three days after birth. Granulopoietic activity declined more gradually after birth. Analysis of the gene expression data revealed the possible involvement of several transcription factors in lineage-specific regulatory mechanisms.

**Conclusion:**

This study enhances our understanding of the postnatal decline of hepatic haematopoiesis in human newborns, highlighting the differential regulation of erythropoiesis and granulopoiesis after birth. These factors bring new in-depth knowledge about the biological processes critical for postnatal adaptation of human newborns.

## 1 Introduction

Many mammalian organs and organ systems begin development prenatally, while significant maturation continues postnatally, with haematopoiesis undergoing a complex transition from embryonic to adult patterns (1). A central role in prenatal haematopoiesis, particularly throughout the 1st and 2nd trimesters (2), is played by the liver, which represents a unique developmental niche allowing haematopoietic stem cells (**HSCs**) to expand massively (on a considerably larger scale in comparison to later haematopoiesis in bone marrow) without developing leukaemia due to unique resilience mechanisms [see e.g., (3)]. HSCs are at the top of a complex haematopoietic differentiation tree of lymphoid and myeloid stem cells as two main branches, with the latter exerting potential for the formation of (i) myeloblasts/granulocytes, (ii) monoblasts/monocytes, (iii) erythropoietic cells, and (iv) megakaryocytes/platelets (4). This hierarchical model is continuously being refined (5–7).

The liver is colonized by several waves of haematopoietic progenitors originating in the yolk sac and aorta-gonad-mesonephros region (2). First signs of haematopoietic activity in the human liver are reported after the gestational week (**GW**) 5 and 8 (8, 9). Based on morphological parameters, prenatal hepatic haematopoiesis in humans was separated into four stages (10), similarly to their original description in mice (11). At its peak during stage III (GW 13–22 in humans, or embryonic days 13–14 in mice), haematopoietic cells can occupy more than 70% of hepatic parenchyma in both species (10, 11). In stage IV (after GW 23 in humans), the haematopoietic activity in the liver is involuting, while HSCs gradually migrate to bone marrow (10). The decline of hepatic haematopoiesis is accelerated after birth as a stress-associated response, possibly due to abrupt elevation of blood oxygen tension (12).

The options to study the final stage of hepatic haematopoiesis in humans are limited, as appropriate samples are extremely scarce (8, 9, 13). To our knowledge, no previous studies characterized in detail individual haematopoietic cell lineages in liver during the critical first hours and days after birth, during the rapid decline of haematopoiesis. To bridge this gap, we took advantage of a unique biobank of autopsy tissue samples from mostly premature newborns. All died within three months, mostly within several hours or days after birth. These materials were used in our previous studies focused on various aspects of perinatal development (13–15), also showing that even in extremely premature newborns, hepatic haematopoiesis is largely absent 7 days after birth (13).

Most recently, we performed RNA sequencing (**RNA-Seq**) of liver, skeletal and heart muscle samples from these newborns, which is intended to be published in short (16) [*n* = 41; data publicly available (17)]. In the present study, the same newborn liver samples previously used for transcriptomic analysis were stained immunohistochemically using established cell type-specific protein markers to quantify haematopoietic subpopulations. Information on the expression of the relevant genes was extracted from whole-organ transcriptome data (17). We aimed to characterize in detail (i) the effect of gestational age at birth (duration of prenatal development; **Gestation**) and postnatal life (length of survival after birth; **Survival**) on hepatic haematopoiesis; and (ii) the variation in the dynamics of postnatal decline in haematopoiesis in human liver, regarding the involvement of individual cell lineages.

## 2 Materials and methods

### 2.1 Liver samples

The unique biobank of autopsies used in this study was obtained within 2–3 h after death from 41 mostly premature newborns born between 20 and 39 weeks of gestation who died shortly after birth. For a characterization of this cohort, see Table 1 and Supplementary Table S1. The detailed analysis of gene expression in liver, heart, and skeletal muscle of these newborns is under preparation (16). Data obtained from the same cohort were also published in our earlier studies (13–15). The results of this study are based on the analysis of only a subset of 25 subjects from the cohort, for which we had histological samples of sufficient quality (see below). At the time of collection (2000–2006), the samples were fixed (3 h) in 3.7% formaldehyde-PBS, dehydrated, and embedded in paraffin for later histological analysis (13) or stored in RNAlater (Ambion, Austin, TX, USA) at −80°C for later transcriptomic analysis in total tissue RNA (16).

**Table 1:** List of cases analysed with basic characteristics [see ref. (17) for full RNA-Seq data].

Case ID	RNA-seq ID (Liver)	Sex	Gestation (weeks)	Birth weight (g)	Survival (days)	Sepsis	Clinical and pathological diagnoses
A87	RNA190	M	28.3	980	2.7	+	PPROM, S, ICH
A90	RNA193	M	24.6	985	17.3	+	RDS, PH, S, ICH
A91	RNA194	M	29.7	830	2.4	−	IA, FGR, ICH
A92	RNA195	M	27.1	820	29.6	−	RDS, RF, ICH
A93	RNA196	M	22.3	500	1.9	−	IA, RDS, ICH
A94	RNA197	M	22.6	450	11.6	+	RDS, S, MOF, ICH
A96	RNA199	M	23.9	700	1.9	−	RDS, ICH
A97	RNA200	F	20.4	350	0	−	LBNS
A98	RNA201	M	32.4	1,840	0.4	−	LBNS, JS, RF
A99	RNA202	F	24.1	320	0	−	LBNS
A100	RNA203	M	22	510	4	−	RDS, ICH
A101	RNA204	F	24.4	580	6.9	−	RDS, PDA, ICH
A102	RNA205	F	24.4	610	20	+	RDS, PDA, BPD, S, ICH
A103	RNA206	F	38.7	1,500	0.8	−	FGR, LH, PH, RF
A105	RNA208	F	26.6	830	36.1	−	RDS, PDA, NEC
A106	RNA209	M	24	620	3.2	+	IA, RDS, S, ICH
A107	RNA210	F	22.7	460	6.7	+	RDS, PDA, NEC, S, ICH
A108	RNA211	M	22.6	590	0.6	−	RDS, ICH
A109	RNA212	M	35.3	1,860	0.1	−	LH, RF
A110	RNA213	M	28.6	1,000	20.6	−	RDS, PDA, NEC, MOF
A111	RNA214	M	27.3	870	3.7	+	RDS, S, ICH
A112	RNA215	F	25.3	700	35	+	RDS, S, M, ICH
A113	RNA216	M	23.4	520	0	−	LBNS, IA, P
A114	RNA217	M	25.7	750	10.9	+	RDS, S, NEC
A115	RNA218	M	32	2,060	1.7	+	RDS, S, ICH

F, female; M, male; BPD, bronchopulmonary dysplasia; FGR, foetal growth restriction; IA, intrauterine asphyxia; ICH, intracranial haemorrhage; JS, Jeune syndrome; LBNS, live-born infant not supported after delivery; M, meningitis; MOF, multiorgan failure; NEC, necrotizing enterocolitis; P, pneumonia; PDA, patent ductus arteriosus; PH, pulmonary haemorrhage; PPROM, preterm premature rupture of membranes; RDS, respiratory distress syndrome; RF, respiratory failure; S, sepsis.

### 2.2 Ethics

Ethics Committee of the General University Hospital, Prague, approved the study (70/18 Grant AZV VES2019 1st Faculty of Medicine, Charles University in Prague). Written informed consent was obtained from the parents.

### 2.3 Transcriptomics

Levels of the transcripts of the genes encoding the protein markers of the individual haematopoietic cell lines were extracted from the RNA-Seq data deposited in the Zenodo repository under accession number 14045261 (16, 17).

### 2.4 Histology and immunohistochemistry

Histological samples showing signs of autolysis were excluded from further analysis. Analyses were performed using 4 µm-thick sections of paraffin-embedded liver samples (2 per each case). For histology, sections were stained using haematoxylin-eosin (HE). In these HE-stained slides, the percentage of haematopoietic cells within the tissue was determined relative to the total number of all cells, primarily hepatocytes.

For the immunohistochemistry, tissue sections were stained using the Ventana BenchMark ULTRA autostainer (Ventana Medical Systems, Tucson, Arizona). Antibodies against CD34 (clone QBEnd/10, Roche), myeloperoxidase (MPO; Rabbit Polyclonal Antibody, Cell Marque), TFRC (CD71; clone 10F11, Bio SB), and ITGB3 (CD61; clone 2f2, Cell Marque) were used. The reactions were visualized by the Ultraview Detection System (Ventana Medical Systems), counterstaining the slides with haematoxylin. The stained slides were dehydrated and covered in a xylene-based mounting medium. All immunohistochemical examinations were assessed by one pathologist and the content was expressed as a fraction of marker-positive cells from all cells in the section.

For the evaluation of the presence of transcription factor GATA1, liver sections were stained by anti-GATA1 antibody (ab181544, Abcam) followed by incubation with biotinylated anti-rabbit secondary antibody (BA-1000, Vector Laboratories), ABC kit (PK6100, Vector Laboratories, Burlingame, USA) and DAB (D4293, Sigma-Aldrich). Counterstaining for nuclei was not performed to prevent interference with nuclear localization of immunohistochemical staining, which prevented the quantification of GATA1^+^ fraction from all cells. Thus, an alternative quantification of GATA1^+^ nuclei per section was performed using semi-automatic analysis in the ImageJ thresholding tool.

### 2.5 Statistical analysis

All values are presented as means ± SEM. Comparisons were judged to be significant at *p* < 0.05 (parameters with 0.1 < *p* < 0.05 are also clearly marked in the respective Figures). Spearman's correlations (r_s_) were calculated for pairs using GraphPad Prism software (v. 10.2.2, 2024, CA). The analysis of the linear mixed-effects model fitted by restricted maximum likelihood (REML) was performed in GraphPad Prism and R environment (v. 4.3.2., 2023) using the lme4 and lmerTest packages. To test the associations between Gestation, Survival, and haematopoietic markers, a multiple linear regression model without interactions was applied. The relationship between Survival/Gestation and a portion of individual lineages was also characterized by the calculation of linear or non-linear regression in GraphPad Prism (R^2^; One-phase decay curve for non-linear regression). Results visualization was performed in GraphPad Prism.

## 3 Results

### 3.1 Cases examined

In total, 25 born-alive infants of both sexes (8 females and 17 males), who all died, primarily due to severe prematurity, were eventually studied (see Materials and Methods; Table 1 and Supplementary Table S1). These newborns differed concerning Gestation (GW 20.4–38.7). Most of the newborns (*n* = 18; 72%) were “extremely preterm newborns” (< GW 28), while only one of them was born at term (≥ GW 37). Some deaths occurred within 24 h (*n* = 7; 28%), but mainly between 1 day and 3 weeks (*n* = 15; 60%), or after approximately one month (*n* = 3; 12%) after birth.

### 3.2 Highly variable content of haematopoietic cells in the newborn's liver with a major contribution of erythropoietic cell line

**Figure 1:**
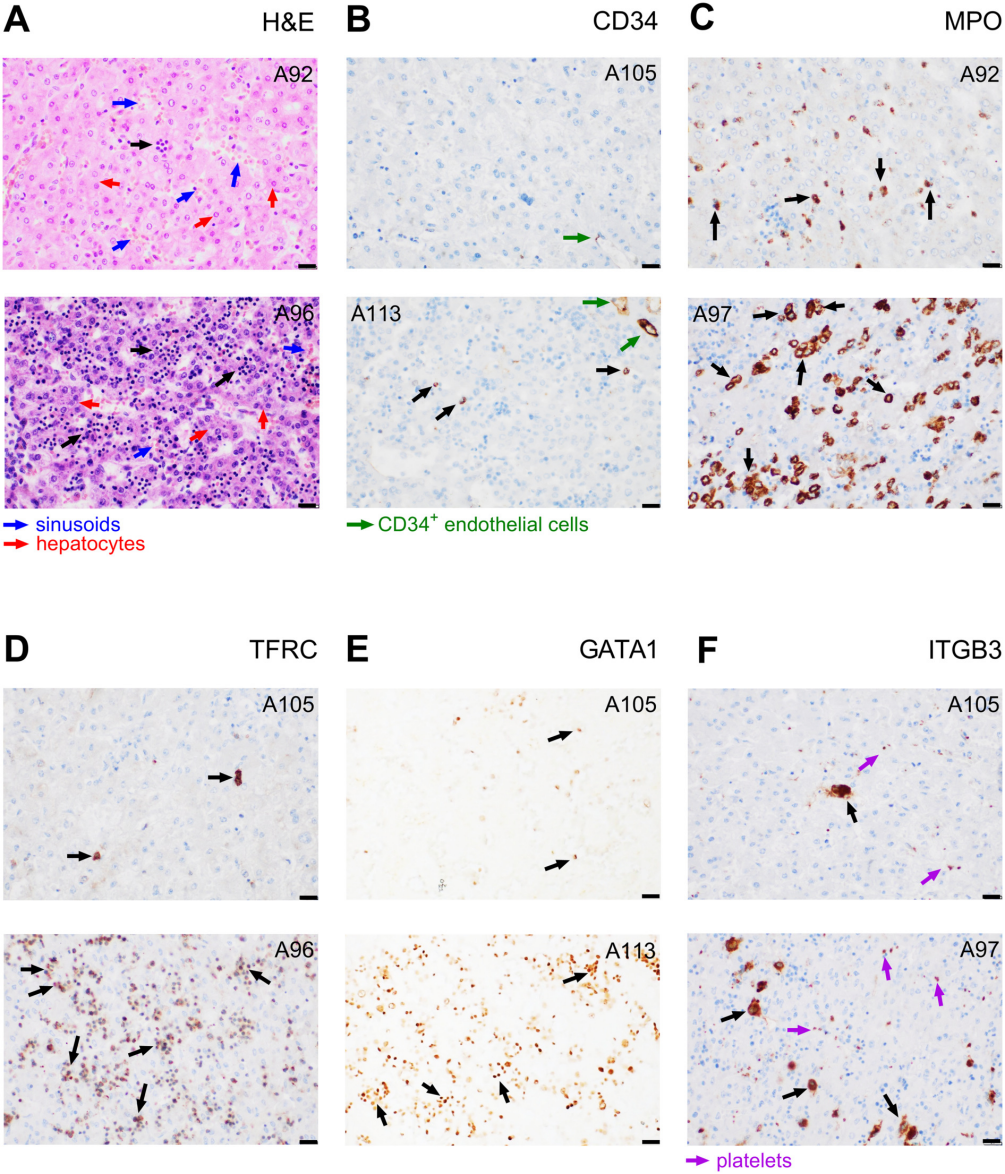
Evaluation of haematopoiesis in liver sections. Samples from subjects exhibiting either a relatively low (upper images) or high (lower images) staining are presented. **(A)** Haematoxylin and eosin staining of haematopoietic cells or their clusters (indicated by black arrows), hepatocytes (larger and less intensely stained nuclei; red arrows), and liver sinusoids (blue arrows). **(B–F)** Immunostaining of protein markers of various cell lineages: **(B)** CD34^+^ haematopoietic and progenitor cells, (black arrows) and endothelial cells (green arrows); **(C)** MPO^+^ myeloblasts (granulopoietic cells; black arrows); **(D)** TFRC^+^ erythropoietic cells (black arrows); **(E)** GATA1^+^ nuclei of erythroid and megakaryocytic cells (black arrows); **(F)** ITGB3^+^ megakaryocytes (black arrows) and platelets (violet arrows). The case sample code number is indicated in the upper corner of each image (see Supplementary Table S1A - Haematopoiesis). Magnification 400×; scale bar represents 20 µm. Gestation and Survival of these subjects were as follows: A92 – Gestational week (GW) 27.1 and Survival 29.6 days; A96 – GW 23.9 and Survival 1.9 days; A97 – GW 20.4 and Survival 0.0 days; A105 – GW 26.2 and Survival 36.1 days; A113 – GW 23.4 and Survival 0.0 days.

The fraction of all haematopoietic cells in the analysed liver sections ranged between 2% and 75% of all cells (Supplementary Table S1A - Haematopoeisis; black arrows in Figure 1A). Individual haematopoietic cell lineages were characterized using immunohistochemical staining of their protein markers, namely: (i) **CD34** (ENSG00000174059; Figure 1B), surface glycophosphoprotein highly expressed on haematopoietic stem and progenitor cells, but to smaller extent also on endothelial cells and small vessels cells (18, 19); (ii) myeloperoxidase (**MPO**, also known as MPX; ENSG00000005381; Figure 1C) expressed in various granulocytes and their precursors, in particular in neutrophils, i.e., a “granulopoietic marker” (20); (iii) transferrin receptor 1 (**TFRC**, also known as CD71; ENSG00000072274; Figure 1D), which is expressed in several cell types, including hepatocytes, but to a particularly large extent in early erythroid precursors during the intermediate normoblast phase, i.e., an “erythropoietic marker” (21); (iv) transcription factor **GATA1** (ENSG00000102145; Figure 1E), important for development of both erythrocytes (22) and platelets (23); and (v) integrin subunit beta 3 (**ITGB3**; also known as CD61; ENSG00000259207; Figure 1F), adhesion receptor on platelets and their progenitors that is essential for normal platelet function (24), i.e., a “megakaryopoietic marker”.

A relatively low number of cells (0.0%–1.3%, mean 0.26%, median 0.20%) was identified as haematopoietic and progenitor cells using CD34 staining (black arrows in Figure 1B). The proportion of granulopoietic MPO-positive (MPO^+^) cells ranged between 1.5 and 29.3% (mean value 9.3%, median 7.5%; Figure 1C). The cells were predominantly localised in clusters. The most abundant lineage in most of the samples (0.4%–72% of all cells, mean value 21.5%, median 12.8%) were TFRC^+^, i.e., erythropoietic cells, again localised predominantly in clusters (Figure 1D). The amount and spatial orientation of GATA1^+^ nuclei visually correspond to that of TFRC^+^ (Figure 1E), suggesting that the observed GATA1 marks a significant proportion of erythropoietic cells. Liver sections contained rather low amounts of ITGB3^+^ megakaryocytes (0%–3.5%, mean 0.96%, median 0.75%), distinguishable from platelets based on their distinct morphology, size, and the presence of the nucleus (Figure 1F).

### 3.3 Stronger influence of Survival than Gestation on hepatic haematopoiesis

**Figure 2:**
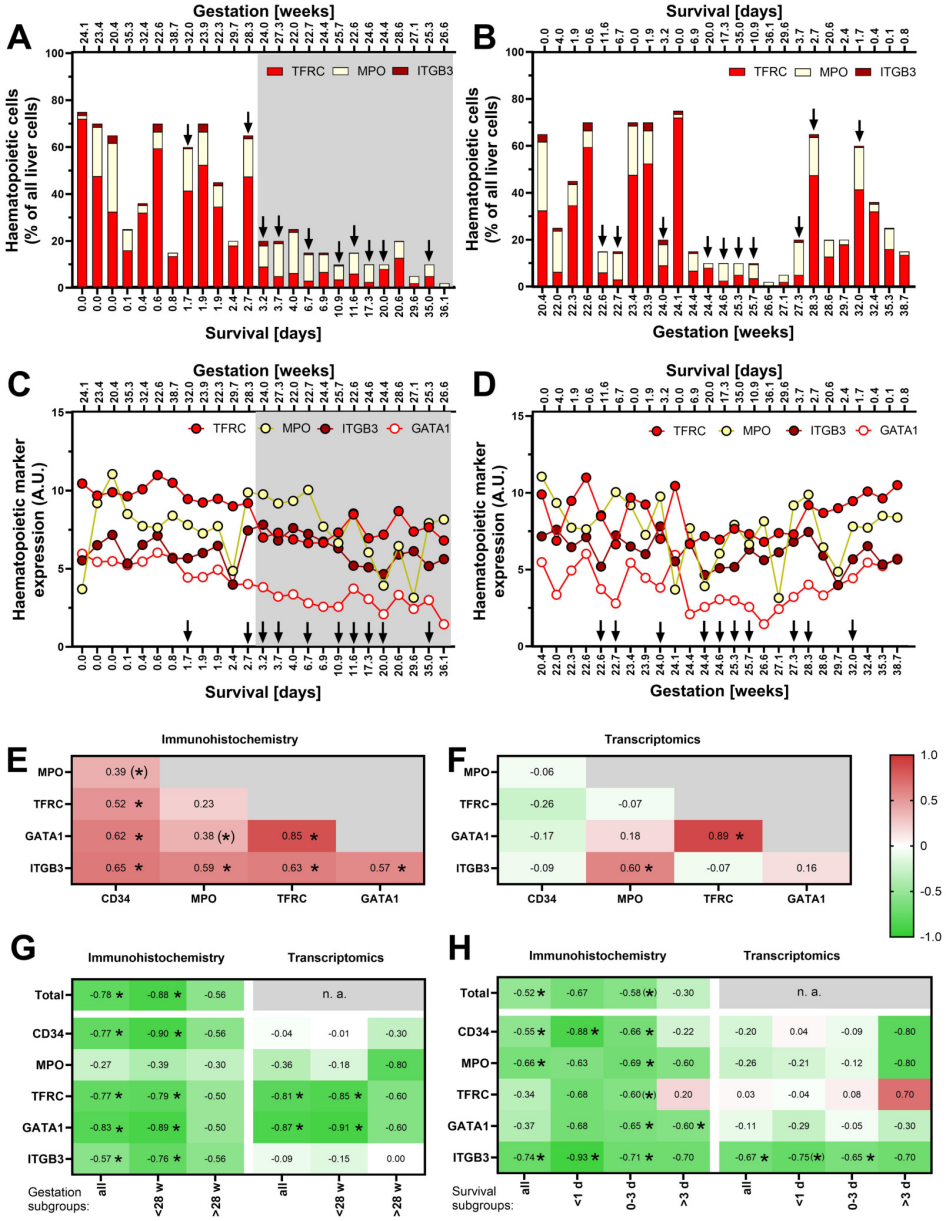
Relationship of hepatic haematopoiesis to Survival and Gestation. **(A)** Relative levels of immunohistochemically identified major components of haematopoiesis in individual newborns ordered by Survival (see Supplementary Table S1A - Haematopoiesis); Survival values are marked on the bottom axis, with Gestation values marked above the panel. In this and the following panels **(A–D)**, Survival and Gestation are marked on the bottom and top axes (the bottom axis always shows the parameter by which the data are ordered). The arrows indicate cases with sepsis (*n* = 10). **(B)** The data as in A in newborns ordered by Gestation. **(C)** Expression levels of genes encoding haematopoietic markers in newborns ordered by Survival. **(D)** The data as in C in newborns ordered by Gestation. **(E)** Mutual correlations between the individual haematopoietic markers analysed by immunostaining; in this and the following panels **(E–H)**, colours and values in each field represent the value of Spearman correlation coefficient (r_s_), * represents significant correlation *p* < 0.05, and (*) represents a correlation with 0.05 < *p* < 0.1). **(F)** Mutual correlations between the expression levels of individual haematopoietic markers analysed by transcriptomics. **(G)** Correlation analysis of Survival and haematopoietic markers (assessed separately in groups of cases with Gestation <28 w, >28 weeks, and all cases together). The analysis includes only cases without diagnosed sepsis (*n* = 15; see Supplementary Figure S1A for corresponding analysis of all cases regardless sepsis). **(H)** Correlation analysis of Gestation and haematopoietic markers (assessed separately in groups of cases with Survival <1 day, 0–3 days, >3 days, and all cases together). The analysis includes only cases without diagnosed sepsis (see Supplementary Figure S1A for corresponding analysis of all cases regardless sepsis).

Gestation and Survival of the newborns are likely to affect the hepatic haematopoietic activity. To explore their effects, the immunohistochemical data above were replotted individually for all newborns in order of increasing Survival (Figure 2A) or Gestation (Figure 2B). Haematopoiesis (especially the relative amount of TFRC^+^ erythropoietic cells) is high in most of the newborns with Survival <3 days, namely in those exhibiting lower Gestation, and it sharply declines postnatally. Thus, the erythropoietic cells do not comprise more than 15% of all cells in any subject surviving >3 days (Figure 2A). On the other hand, the strong effect of Survival mostly masks any potential effect of Gestation in this type of visualisation (Figure 2B).

To enhance our ability to characterise hepatic haematopoiesis using samples from this unique newborn cohort, we decided to incorporate RNA-Seq data analysis into our analytical approach. In general, quantification of immunohistochemical staining of the described protein markers closely correlated with the expression of the respective genes (Spearman's correlation coefficient, **r_s_**, in the range of 0.65–0.87; *p* < 0.05; not shown). Only in the case of CD34, the least expressed of the analysed markers, there was no significant correlation (not shown). Similarly to the immunohistochemical results described above, the RNA-Seq data indicate a decline in erythropoietic markers after birth (Figure 2C). This trend is illustrated not only by a lower expression of the gene encoding the classical TFRC marker, but also by a drop in the expression of the gene for the transcription factor GATA1. The effect of Gestation on the expression of haematopoietic gene markers is not immediately apparent (Figure 2D).

Ongoing infections can potentially affect the level of haematopoiesis, particularly granulopoiesis. In Figures 2A–D, we thus highlighted (by arrows) the cases with diagnosed sepsis. There were 10 such cases out of the 25 cases. Although we did not observe any clear effect of sepsis on haematopoietic markers in these graphs, we decided to perform the following analyses in parallel on a subgroup of cases without sepsis and on all samples regardless sepsis (these are shown in Supplementary Figure S1).

Taken together, the above data document a substantial decline in hepatic haematopoiesis in the first days after birth, which is influenced by Gestation but without a clear effect of the presence or absence of sepsis.

### 3.4 Distinct patterns of postnatal haematopoietic lineages development

Inspection of Figures 2A–D suggested that the dynamics of postnatal suppression of hepatic haematopoiesis may differ among the individual cell lineages. Therefore, we tested mutual correlations of various cell type markers throughout the cohort using the immunohistochemical data (Figure 2E). A particularly strong correlation between TFRC and GATA1, which are both expressed in cells of erythroid lineage, was observed (r_s_ = 0.85). Relatively high (r_s_ > 0.5) were also the mutual correlations between all other markers, except of the granulopoietic marker MPO, which correlated significantly only with the megakaryopoietic marker ITGB3. Fewer correlations were found at the transcript level (Figure 2F), confirming the correlations between TFRC and GATA1 and between MPO and ITGB3. The above data suggest that erythropoiesis and megakaryopoiesis decline with similar dynamics, while the decline of granulopoiesis differs from the others.

To further characterize the specific patterns of postnatal development of various haematopoietic cell lineages, correlations between Survival and each of the lineage markers were expressed by coefficient r_s_. We applied this approach to all the studied subjects together and separately on subgroups of subjects with Gestation < GW 28 (extremely preterm newborns; *p* = 18) and newborns with longer prenatal development (> GW 28; *n* = 7). Immunohistochemical staining revealed a negative correlation of all cell types except MPO^+^ cells (i.e., granulopoietic cell lineage), for both (i) all the cases analysed together, and (ii) cases with < GW 28 (but not > GW 28; Figure 2G and Supplementary Figure S1A). These differences among granulopoietic, erythropoietic (marked by both TFRC and GATA1), and ITGB3^+^ megakaryopoietic cell lineages were consistent with the results of transcriptomic analysis, although this analysis showed no correlation with expression of CD34 gene (haematopoietic and progenitor cells; Figure 2G and Supplementary Figure S1A).

Next, we sought to correlate also Gestation with the markers of various cell lineages. Considering the strong effect of Survival (above), we assessed the relationships separately within various subgroup of newborns, i.e., in those with Survival <1 day, 0–3 days, and >3 days (Figure 2H and Supplementary Figure S1B). According to immunohistochemistry, a portion of haematopoietic stem and progenitor cells (CD34^+^) and megakaryocytes (ITGB3^+^) negatively correlated with Gestation in the group with Survival <1 day. The number of cells of granulopoietic and megakaryopoietic lineages correlated with Gestation if all the subjects were analysed together. Surprisingly, the erythroid transcription factor GATA1 was correlated with Gestation only in the group with Survival 0–3 days and >3 days. Only ITGB3 was correlated to Gestation at the transcript level (Figure 2H).

Taken together, these results confirmed that hepatic haematopoiesis is negatively related to both Survival and Gestation. They unequivocally demonstrated distinct patterns of postnatal haematopoietic lineages development.

### 3.5 Gestation and Survival together explain up to 30%–60% of the variance in hepatic haematopoiesis

**Figure 3:**
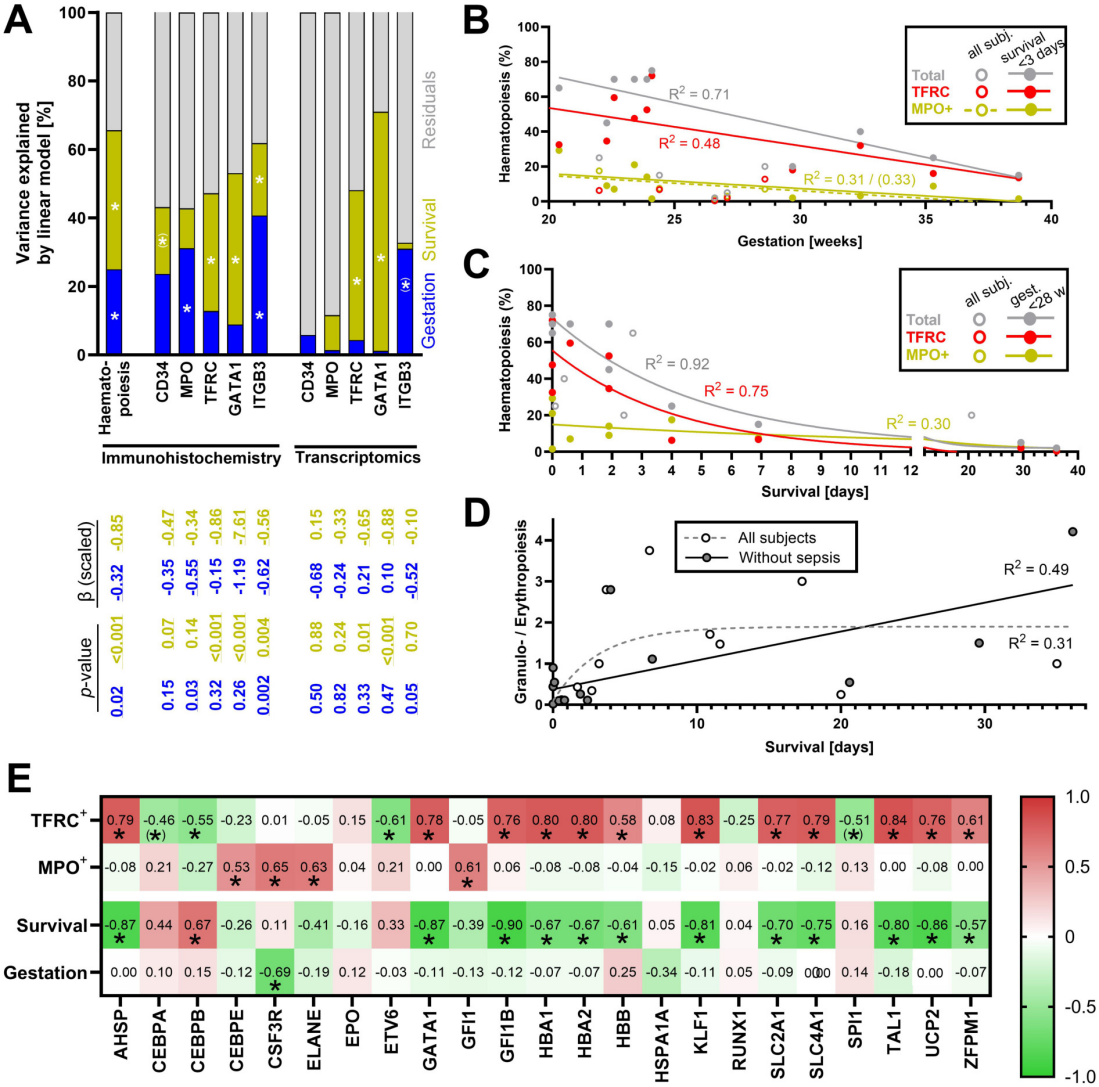
Combined effect of Gestation and Survival on various components of haematopoiesis. Only cases without diagnosed sepsis were analysed in this figure (except of **D**); for the corresponding analyses with all cases including those with diagnosed sepsis, see Supplementary Figures S1C,F. **(A)** Variance in individual parameters explained by multiple linear regression model of Survival and Gestation without interactions. The table below the columns shows scaled beta values and *p*-values of colour-coded components of the model. The percentage of variance explained by each factor was extracted from the model. The scaled beta coefficient represents a standardized estimated change in the response variable per unit of change in the predictor variable. *P-*values < 0.05 were considered significant. * represents a significant correlation *p* < 0.05, and (*) represents a correlation with 0.05 < *p* < 0.1). **(B)** Linear regression of overall haematopoiesis, erythropoiesis, and granulopoiesis (all assessed by immunohistochemistry) throughout Gestation (analysis performed separately for all newborns and only for newborns with Survival <3 days). R^2^ is shown for significantly non-zero regression lines (*p* < 0.05, except for MPO^+^ positive cells in subjects with Survival <3 days). In the case of regression of MPO^+^ cells, R^2^ = 0.31 (*p* = 0.03) for all subjects and R^2^ = 0.33 (*p* = 0.08) for subjects with Survival <3 days. **(C)** Non-linear regression (one-phase decay curve) of total haematopoiesis, erythropoiesis, and granulopoiesis (all assessed by immunohistochemistry) and Survival (regression curves and R^2^ are shown only for subjects < GW 28). **(D)** Non-linear regression analysis of Survival and ratio between erythropoietic and granulopoietic cells in all newborns (grey dashed curve), and in the newborns without diagnosed sepsis (black curve). **(E)** Correlations between the expression of regulatory and metabolic genes on the one hand, and erythropoiesis, granulopoiesis, Survival, and Gestation on the other hand; colours and values in each field represent the value of Spearman correlation coefficient (r_s_), * represents a significant correlation *p* < 0.05, and (*) represents a correlation with 0.05 < *p* < 0.1).

To dissect the differential effects of Survival and Gestation on postnatal changes of the global haematopoietic activity in the liver, as well as various cell lineages involved, multiple linear regression analysis was performed (Figure 3A; compare to Supplementary Figure S1C showing all cases regardless of sepsis). Based on the immunohistochemical data, the effect of Survival on the global haematopoiesis is stronger, explaining 11%–44% of the variance in the individual parameters (Figure 3A). Adding the effect of Gestation to the multilinear model allows us to explain 40%–65% of the variance. The effect of Gestation is significant in the case of MPO and ITGB3, while it is completely absent in the case of GATA1.

In the case of transcriptomic data, multilinear regression provides variable outcomes ranging from < 10% of explained variance in CD34 expression to 70% of explained variation in GATA1 expression (Figure 3A). This is mostly due to the effect of Survival. Only the variance in ITGB3 is partially explained by the variability in Gestation.

Thus, if assessed by immunohistochemistry, the suppression of all haematopoietic lineages can be largely explained by Survival with the smaller contribution of Gestation. The relationships are less clear if haematopoiesis is assessed by the transcriptomic analysis.

### 3.6 Faster decline in erythropoiesis than granulopoiesis

Correlation analysis of immunohistochemical data (Figure 2H) revealed different dynamics between the decline of hepatic granulopoiesis and other components of haematopoiesis. In subjects with Survival <3 days, both overall haematopoiesis, erythropoiesis, and granulopoiesis are strongly linearly related to Gestation (with a coefficient of determination R^2^ = 0.71, R^2^ = 0.48 and R^2^ = 0.33, respectively; Figure 3B, see also Supplementary Figure S1D for analyses of all cases regardless of sepsis). A striking difference between the regulations of erythropoiesis and granulopoiesis is observed right after birth (Figure 3C, see also Supplementary Figure S1E). Erythropoiesis accounts for a large proportion of overall haematopoiesis especially on the first day after birth but declines rapidly thereafter. On the other hand, granulopoiesis, which at the beginning represents only less than 30% of the overall haematopoiesis, slowly decreases, almost linearly, and the proportion of granulopoietic cells among all haematopoietic cells thus increases. This phenomenon is illustrated by a rise in the ratio between granulopoietic and erythropoietic cells (Figure 3D), which rises fast on the first day after birth and slows down later. Survival can explain up to 49% of the variance in this parameter in subjects without sepsis. Thus, erythropoietic cells represent the most abundant haematopoietic lineage in the liver throughout Gestation. At the same time, erythropoiesis is suppressed quickly after birth, resulting in a relative increase in the proportion of granulopoietic cells. Hepatic granulopoiesis in the last trimester seems less affected by Gestation and decreases more slowly after the birth.

### 3.7 Transcriptional control explains distinct patterns of postnatal development of erythropoiesis and granulopoiesis

To characterize the mechanisms underlying the differential control of erythropoiesis and granulopoiesis in postnatal human liver, we analysed correlations between the number of cells positive for protein markers of these lineages (i.e., TFRC and MPO, omitting GATA1 – a transcription factor of erythropoiesis which was already analysed – see above) and transcripts of genes encoding selected transcription factors and regulators involved in the control of haematopoiesis, as well as selected gene markers of their activity (Figure 3E; compare to Supplementary Figure S1F for all cases regardless of sepsis; full list of analysed transcripts in Supplementary Table S1B – Regulation). We found that the number of TFRC^+^ cells closely correlates (r_s_ > 0.7, *p* < 0.0001) with the expression of transcription factors *GFI1B* (25), *AHSP* (encoding ERAF), *KLF1* (26), and *TAL1* (TAL1/SCL) (27) [Figure 3E, Supplementary Figure S1F, Supplementary Table S1B and the gene expression data in the external Zenodo database (17)]. In contrast, the number of MPO^+^ cells correlated with expression of transcription regulators *CEBPE*, *CSF3R*, *ELANE*, and *GFI1* (28).

In addition, the expression of genes encoding proteins that have been shown to be linked to haematopoiesis in the newborn liver was also considered, i.e., haemoglobin genes *HBA1*, *HBA2*, and *HBB*, and other genes such as *UCP2* (13) and glucose transporter *SLC2A1* (encoding GLUT1) (29). The expression of all these genes strongly correlates with the number of TFRC^+^ cells (i.e., *HBA1*, *HBA2*, *HBB*, *UCP2*, *SLC2A1*; Figure 3E and Supplementary Figure S1F).

The expression of several of highlighted transcription factors and other markers also closely and negatively correlates with Survival (r_s_ between −0.7 and −0.9, *p* < 0.0001; specifically: *AHSP*, *GFI1B*, *KLF1*, *TAL1*, *HBA1*, *HBA2*, *HBB*, *UCP2*, *SLC2A1*; Figure 3E and Supplementary Figure S1F), suggesting that they could be used as alternative transcriptomic markers of hepatic erythropoiesis equally well or better than the traditional histological marker TRFC.

## 4 Discussion

Here, we provide insights into the dynamics of hepatic haematopoiesis in human newborns, utilizing a unique biobank of samples and employing both histological and transcriptomic approaches. Our findings reveal several key aspects of this process, namely: (i) fast postnatal decline of hepatic haematopoiesis; (ii) gestational age effects; and (iii) lineage-specific dynamics.

We observed a significant inverse relationship between Survival and the level of hepatic haematopoiesis. The decline in haematopoiesis is particularly pronounced during the first three days of life, when it drops to half its original level within a short period after birth, regardless of the gestational age. This rapid decline aligns with some previous observations (9, 12), and extends our earlier findings (13). We have also noticed the effect of Gestation on hepatic haematopoiesis. This effect is partially masked by the strong influence of Survival. Analysis in newborns surviving for less than 3 days suggests a gradual prenatal decline of haematopoiesis throughout the third trimester. This is clearly apparent, especially in the case of more abundant erythropoietic lineage and thus supports the proposed staged model of hepatic haematopoiesis (10, 11).

Our results demonstrate distinct patterns of decline among different haematopoietic lineages, i.e., (i) erythropoiesis, the most abundant lineage, shows a rapid, exponential decrease after birth, which is consistent with the hypothesis that the abrupt elevation of blood oxygen tension plays a crucial role in suppressing erythropoiesis, as suggested earlier (12); (ii) granulopoiesis exhibits a slower, more linear decline, leading to an increasing ratio of granulopoietic to erythropoietic cells over time (this differential regulation has not been previously reported in the context of neonatal hepatic haematopoiesis); and (iii) megakaryopoiesis appears to decline rapidly after birth, similarly to erythropoiesis. This is in agreement with the shared regulatory mechanisms of these lineages (30), nevertheless, megakaryopoiesis represented a rather negligible portion of haematopoiesis in all the samples.

Considering the proposed role of blood oxygen tension in suppression of erythropoiesis (12), it would be interesting to compare level of erythropoiesis between left and right liver lobe which are differentially perfused by umbilical venous and portal venous blood (31). Unfortunately, we do not have exact information about the lobes which the samples in our cohort were collected from and we thus cannot confirm the hypothesis by such evidence.

It is to be inferred that the observed faster decline in hepatic erythropoiesis compared to granulopoiesis serves as a compensatory mechanism for the vastly different lifespans of these cell types in circulation. This differential decline balances the rapid turnover of granulocytes, which have a half-life of hours to days, against the prolonged lifespan of erythrocytes. By adjusting production rates, the liver may maintain appropriate levels of both cell types in the bloodstream despite their contrasting longevities. This holds even for preterm newborns exhibiting relatively short erythrocyte lifespan (approximately 50 days) compared with approximately 120 days in adults (32). Moreover, neonatal infection, which was common in the premature neonates in this cohort (13–16), would also affect the granulocyte production and prolong the presence of haematopoietic cells in the liver. Nevertheless, excluding samples with sepsis did not change the overall conclusions of our analyses.

The rapid decline in erythropoiesis after birth, which contrasted with the slower decline of granulopoiesis, suggests differential postnatal regulation of these lineages. Building on our previously performed characterisation of the hepatic transcriptome of the cases analysed here (see Materials and Methods), we confirmed the involvement of specific transcription factors and regulators (*AHSP*, *GATA1*, *GFI1B*, *KLF1*, and *TAL1*) in the postnatal erythropoiesis. The expression of these regulatory proteins, as well as some other markers (e.g., *SLC2A1*, *UCP2*, *HBA1*, *HBA2*), closely correlates with Survival and can thus be potentially used as an alternative transcriptomic marker to TFRC. On the other hand, a slower decline of granulopoiesis corresponded to the expression of *CEBPE*, *CSF3*, *ELANE*, and *GFI1*.

In preterm newborns without sufficiently developed haematopoietic bone marrow, the fast decline in hepatic haematopoiesis after birth can result in severe anaemia frequently observed in these infants. Further studies of the mechanisms controlling hepatic haematopoiesis in newborns are required to understand the causes of changes in red blood cell count (33–35), neutrophilia (12, 36), and thrombocytopenia (37, 38) in these infants. They could also be instrumental in improving strategies for *ex vivo* blood cell generation, an area of growing interest in regenerative medicine (3).

Our study also highlights the strengths and limitations of histological and transcriptomic approaches. Immunohistochemistry provided more consistent results regarding the relationships between haematopoietic markers, Survival, and Gestation than transcriptomic analysis. The limitations of this study include potential bias caused by the use of post-mortem samples and the inability to perform single-cell transcriptomics (39) due to the nature of the archived samples. Future research using these advanced techniques on fresh samples could provide even more detailed insights into the cellular dynamics of hepatic haematopoiesis, as has been demonstrated in other haematopoietic contexts (4).

In summary, this study provides fundamental insights into the postnatal decline of hepatic haematopoiesis in human newborns, revealing distinct regulatory patterns for erythropoiesis and granulopoiesis after birth. Our findings demonstrate that hepatic haematopoiesis remains influenced by gestational age for up to three days postpartum. While bringing new in-depth knowledge about the biological processes critical for newborns' survival, our results may have implications for neonatal care.

## Data Availability

The datasets presented in this study can be found in online repositories. The names of the repository and accession number can be found in Stranecky and Kopecky (17), selected supporting information can be found in Supplementary Material.

## Supplementary material

Supplementary Figure S1 Analyses performed, including all cases regardless of sepsis (*n* = 25), corresponding to individual figures showing analyses on cases without diagnosed sepsis. **(A)** (corresponds to Figure 2G) Correlation analysis of Survival and haematopoietic markers (assessed separately in groups of cases with Gestation <28 and >28 weeks, and all cases together). **(B)** (corresponds to Figure 2H) Correlation analysis of Gestation and haematopoietic markers (assessed separately in groups of cases with survival <1 day, 0-3 days, >3 days, and all cases together). The analysis includes only cases without diagnosed sepsis [see **(A)** for corresponding analysis of all cases regardless sepsis]. **(C)** (corresponds to Figure 3A) Variance in individual parameters explained by multiple linear regression model of Survival and Gestation without interactions. The table below the columns shows scaled beta values and *p*-values of colour-coded components of the model. The percentage of variance explained by each factor was extracted from the model. The scaled beta coefficient represents a standardized estimated change in the response variable per unit of change in the predictor variable. *p*-values <0.05 were considered significant and are marked with asterisks. **(D)** (corresponds to Figure 3B) Linear regression of overall haematopoiesis, erythropoiesis, and granulopoiesis (all assessed by immunohistochemistry) throughout Gestation (analysis performed separately for all newborns and only for newborns with Survival <3 days). R^2^ is shown for significantly non-zero regression lines (*p* < 0.05). **(E)** (corresponds to Figure 3C) Non-linear regression (one-phase decay curve) of total haematopoiesis, erythropoiesis, and granulopoiesis (all assessed by immunohistochemistry) and Survival (regression curves and R^2^ are shown only for subjects with Gestation <28 weeks). **(F)** (corresponds to Figure 3E) Correlations between the expression of regulatory and metabolic genes on the one hand, and erythropoiesis, granulopoiesis, Survival, and Gestation on the other hand; colours and values in each field represent the value of Spearman correlation coefficient, * represents a significant correlation *p* < 0.05, and (*) represents a correlation with 0.05 < *p* < 0.1).
