# Supplementary figures and images for: Faster postnatal decline in hepatic erythropoiesis than granulopoiesis in human newborns
Source: Front Pediatr. 2025 May 20;13:1572836. doi: 10.3389/fped.2025.1572836 (PMC12129751; doi:10.3389/fped.2025.1572836)

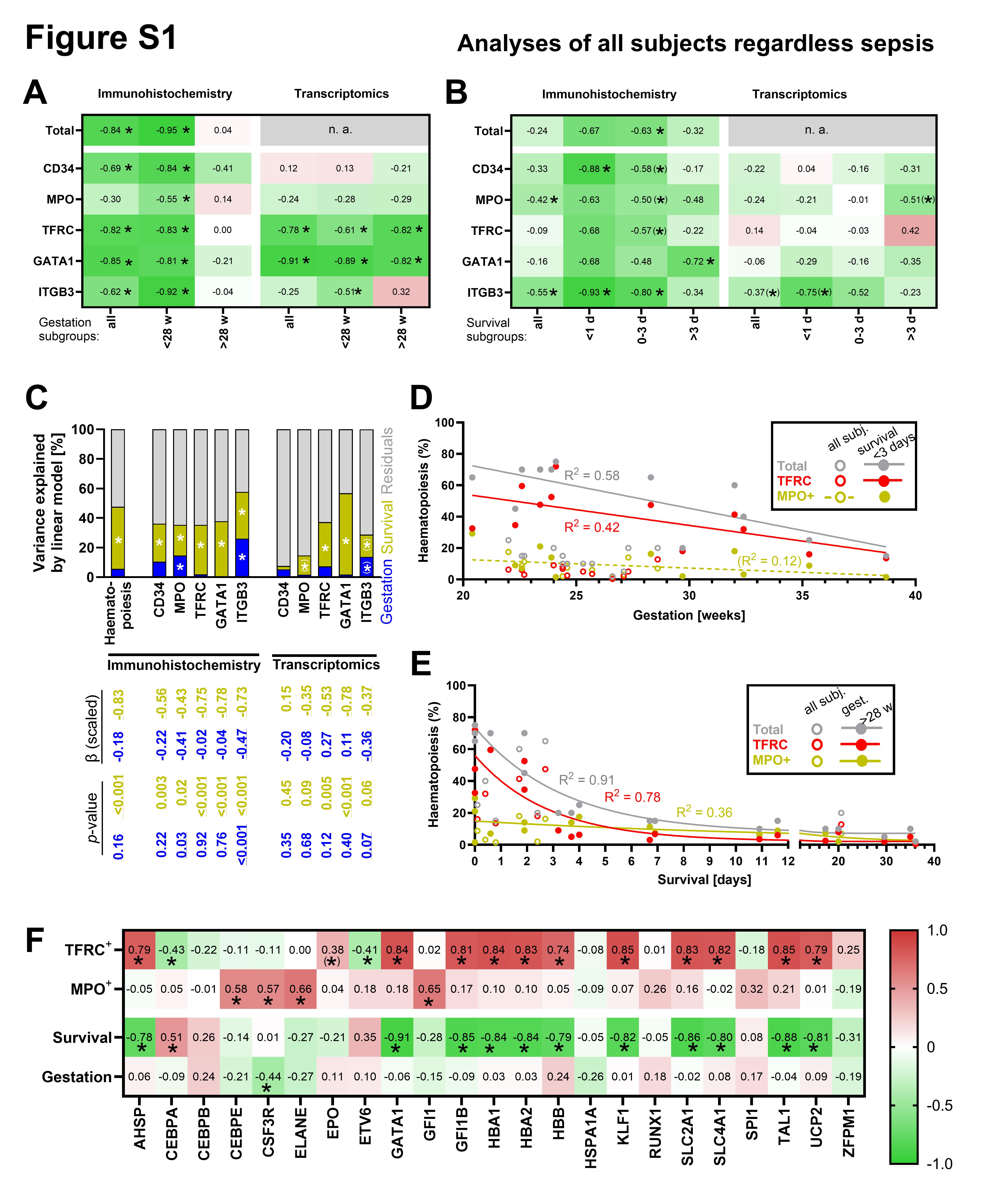

Supplement: Supplementary Figure S1 — Analyses performed, including all cases regardless of sepsis (n = 25), corresponding to individual figures showing analyses on cases without diagnosed sepsis. (A) (corresponds to Figure 2G) Correlation analysis of Survival and haematopoietic markers (assessed separately in groups of cases with Gestation <28 and >28 weeks, and all cases together). (B) (corresponds to Figure 2H) Correlation analysis of Gestation and haematopoietic markers (assessed separately in groups of cases with survival <1 day, 0-3 days, >3 days, and all cases together). The analysis includes only cases without diagnosed sepsis [see (A) for corresponding analysis of all cases regardless sepsis]. (C) (corresponds to Figure 3A) Variance in individual parameters explained by multiple linear regression model of Survival and Gestation without interactions. The table below the columns shows scaled beta values and p-values of colour-coded components of the model. The percentage of variance explained by each factor was extracted from the model. The scaled beta coefficient represents a standardized estimated change in the response variable per unit of change in the predictor variable. p-values <0.05 were considered significant and are marked with asterisks. (D) (corresponds to Figure 3B) Linear regression of overall haematopoiesis, erythropoiesis, and granulopoiesis (all assessed by immunohistochemistry) throughout Gestation (analysis performed separately for all newborns and only for newborns with Survival <3 days). R2 is shown for significantly non-zero regression lines (p < 0.05). (E) (corresponds to Figure 3C) Non-linear regression (one-phase decay curve) of total haematopoiesis, erythropoiesis, and granulopoiesis (all assessed by immunohistochemistry) and Survival (regression curves and R2 are shown only for subjects with Gestation <28 weeks). (F) (corresponds to Figure 3E) Correlations between the expression of regulatory and metabolic genes on the one hand, and erythropoiesis, granulopoiesis, Survi [file Image1.jpg]
